# Digital cognitive stimulation in elderly breast cancer patients: the Cog-Tab-Age feasibility study

**DOI:** 10.1186/s12906-024-04507-0

**Published:** 2024-05-31

**Authors:** Giulia Binarelli, Marie Lange, Mélanie Dos Santos, Mylène Duivon, Aurélie Capel, Marie Fernette, Antoine Boué, Jean-Michel Grellard, Laure Tron, Djihane Ahmed-Lecheheb, Bénédicte Clarisse, Olivier Rigal, Johan Le Fel, Florence Joly

**Affiliations:** 1 Clinical Research Department, Centre François Baclesse, Caen, 14076 France; 2 ANTICIPE U1086 INSERM-UCN, Equipe Labellisée Ligue Contre le Cancer, Centre François Baclesse, Normandie Université UNICAEN, Caen, 14000 France; 3 Services Unit PLATON, Cancer and cognition Platform, University of Caen Normandy, Caen, 14000 France; 4 University Hospital of Caen, Caen, 14000 France; 5 CHU Grenoble Alpes, VOIRON, 38500 France; 6 Care Support Department, Centre Henri Becquerel, Rouen, France; 7 Medical Oncology Department, Centre Henri Becquerel, Rouen, France

**Keywords:** Digital intervention, Cancer-related cognitive impairment, Elderly, Cognitive stimulation, Breast cancer, Quality of life

## Abstract

**Background:**

Elderly cancer patients often experience cognitive difficulties that can affect their quality of life and autonomy. However, they are rarely included in clinical trials, and only one study has explored the feasibility of cognitive training in this population. While digital cognitive training has been successful in improving cognition in younger patients, its feasibility in elderly patients requires evaluation.

**Objectives:**

This feasibility study primarily focused on evaluating patients’ ability to use digital cognitive stimulation (usability). Secondary objectives were to evaluate acceptability, adherence, and satisfaction with regard to digital cognitive stimulation in elderly breast cancer patients.

**Methods:**

Elderly breast cancer patients at least 70 years old who were receiving cancer treatment (chemotherapy, targeted therapy, and/or radiotherapy) were recruited. Cognitive complaints were evaluated at baseline using the Functional Assessment of Cancer Therapy-Cognitive Function scale (FACT-Cog). Participants were invited to attend three 20-minute sessions of digital cognitive stimulation using HappyNeuron PRESCO software App on tablets, with the first session being supervised by a neuropsychologist and the two others being performed independently either at home or at the cancer center. We hypothesized that participants would spend 10 of the 20 min of the given time with the tablet completing exercises (training time). Thus, the usability of digital cognitive stimulation was defined as completing at least three exercises during the training time (10 min) of one of the two training sessions in autonomy. The proportion of patients who agreed to participate (acceptability) and completion of planned sessions (adherence) were also estimated. Satisfaction was evaluated post-intervention through a self-report questionnaire.

**Results:**

240 patients were initially screened, 60% (*n* = 145) were eligible and 38% agreed to participate in the study. Included patients (*n* = 55) had a mean age of 73 ± 3 years, 96% an ECOG score of 0–1 and were undergoing radiotherapy (64%), and/or chemotherapy (47%) and/or targeted therapy (36%) for stage I-II breast cancer (79%). Most patients reported significant cognitive complaints (82%) and 55% had previous experience with digital tools (*n* = 30). The usability rate was 92%, with 46 out of 50 evaluable participants completing at least three exercises during the training time. The adherence rate was 88%, with 43/50 participants completing all planned sessions. Participants were largely satisfied with the cognitive intervention format (87%). They preferred to complete sessions at the cancer center under the supervision of the neuropsychologist than alone at home (90%).

**Conclusions:**

The high level of usability, adherence and satisfaction in this study shows for the first time the feasibility of digital cognitive stimulation in cancer patients older than 70 years. However, the intervention should be proposed only to patients reporting cognitive complaints and should be structured and supervised to improve acceptability and adherence.

**Trial Registration:**

ClinicalTrials identifier: NCT04261153, registered on 07/02/2020.

## Introduction

### Cognitive impairment in elderly patients with cancer

Cognitive impairment is highly associated with age, with almost 14 million adults aged 65 or older in the United States who will experience cognitive impairment by 2060 [1].

Cancer is also an age-related disease, with about 70% of cancer patients older than 65 years [2]. Owing to the general increase in life expectancy and the development of more efficient cancer therapies, elderly cancer patients are living longer and in better health [3]. Nevertheless, elderly patients are at greater risk of the side-effects of cancer treatments, including cognitive decline. This decline, known as cancer-related cognitive impairment (CRCI) and more recently termed cancer-related cognitive decline, may be more prevalent in elderly cancer patients (up to 49%) than in younger ones (20–30%) [4, 5]. This high prevalence is thought to be related to the accelerated brain aging induced by cancer itself and its treatments [5, 6]. The most affected cognitive domains are processing speed, executive functions, and memory [7, 8].

The severity of CRCI can be influenced by several factors, such as chemotherapy treatments [9], comorbidities, pain and prior cognitive difficulties [10, 11]. Cognitive impairment in elderly cancer patients has been associated with functional decline [12], malnutrition [10, 13], frailty [14] and lower survival rates [15]. In addition, CRCI can affect patients’ quality of life and their autonomy [12, 16], medical decisions, increase the risks of complications and make medical care and treatment compliance more challenging [17–19]. This is especially important given the current rise in the prescription of oral oncology treatments such as hormone therapy and targeted therapies.

### Cognitive interventions for CRCI in elderly patients with cancer

Elderly patients with cancer are often overlooked in clinical trials [20] as well as in interventional studies for CRCI. To our knowledge, only one feasibility study has investigated the acceptability, usability, and adherence of a digital cognitive intervention among 60 patients with prostate cancer with a mean age of 66 years [21]. Participants were randomly assigned to either 8 weeks of computerized cognitive training (using BrainHQ software) or usual care.

Compared to usual care, computerized cognitive training improved reaction time but had an unfavorable effect on memory. Overall, patients were satisfied with the digital program.

### Digital interventions in the elderly

According to a systematic review on healthy elderly adults, digital cognitive interventions such as cognitive software and video games are either comparable or superior to paper-and-pencil interventions in improving cognition [20]. In addition, in most studies, patients did not require significant technological skills to participate in the program. Although many elderly patients expressed anxiety regarding the use of unfamiliar technology at the beginning of the training, most of them reported satisfaction at the end [22]. Some participants viewed learning to use video games as a beneficial mental exercise, while others felt that it improved their relationship with their grandchildren [22].

Digital cognitive interventions are gaining attention for the management of CRCI in young breast cancer patients and have exhibited promising results [23, 24]. High adherence rates (65–95%) were found for digital cognitive interventions, with moderate to high satisfaction levels [23, 24]. The engaging nature of digital interventions, with enjoyable interactive exercises, automatic email reminders, and personalized difficulty levels that adapt to users’ performances, partially account for their success. However, information on digital interventions for elderly cancer patients with CRCI is limited [23], with only the study previously cited concerning a digital cognitive intervention [21].

To assess the efficacy of digital interventions for managing CRCI in the elderly, several parameters should be considered in preliminary studies. These include determining the acceptability of digital interventions, understanding patient preferences for intervention type (such as alone and home-based or onsite with supervision), identifying barriers and facilitators for implementing such interventions in clinical practice, and assessing patients’ ability to use digital devices. For these reasons, we designed a feasibility study to document these aspects regarding digital interventions for managing CRCI in elderly breast cancer patients.

### Objectives

To investigate the feasibility of digital interventions among elderly breast cancer patients, the primary objective of this study was to assess the usability of the HappyNeuron PRESCO software App on tablets in elderly patients with breast cancer. Secondary objectives were to assess eligibility, acceptability, adherence, and satisfaction with regard to a digital cognitive intervention in elderly breast cancer patients.

## Methods

### Study design and population

This was a longitudinal bi-center feasibility study consisting of a non-randomized experimental intervention focused on elderly patients with breast cancer. The intervention under consideration was a digital cognitive intervention using the HappyNeuron PRESCO software App on tablets. Participants with breast cancer aged 70 years and older, undergoing treatment with chemotherapy, targeted therapy or radiotherapy, regardless of cancer stage, were recruited by a neuropsychologist at the Comprehensive Cancer Centers in Caen and Rouen (France). Non-eligibility criteria included (a) neurological and/or psychological conditions, (b) the presence of brain metastases, (c) previous treatment with brain radiotherapy, (d) documented alcohol or drug abuse or medical conditions which could impact their ability to participate, (e) having severe visual and/or hearing impairment, (f) not speaking French, and (g) screening positive for overall cognitive impairment based on the Montreal Cognitive Assessment (MoCA) score [25] (cutoff based on the patient’s age and education level, according to GRECOGVASC normative data [26]). All patients included in the study provided informed written consent.

### Intervention

#### Tool: the happyneuron presco software app

The HappyNeuron PRESCO software® is a digital cognitive therapy tool. For this study, it was installed in the app format on a tablet and for use offline. By creating an account, the user’s progress can be recorded in the databases. Developed by a neurologist, this software is designed to improve 12 cognitive domains, including attention, processing speed, memory, and executive functions, which are common in CRCI. The software offers 41 exercises divided into nine levels of difficulty and is available in 11 different languages. This software has been used in numerous studies in the field of mental health and in two studies with young cancer patients, with positive results on cognition [27–29].

Users are free to choose from the 41 exercises and levels of difficulty, or the therapist can customize the training session by selecting the cognitive domains to be trained, the level of difficulty, and the session duration. Prior to starting the exercises, a practice example is available. Upon completion of each exercise, the software provides automatic feedback to praise or motivate the participant to persist despite any setbacks.

### Procedure

After inclusion, participants were asked to use a tablet to complete three 20-minute sessions on the digital cognitive stimulation based on the software HappyNeuron. The first session was performed in the presence of a neuropsychologist while the other two sessions were performed in autonomy without the neuropsychologist’s help. Participants met the neuropsychologist for the first session in a quiet room at the cancer center. During the first session, they were given a tablet, which they would use until the end of their participation in the study. During this session, the neuropsychologist demonstrated several exercises (always at level one of difficulty) to the participants (Table 1), helped them navigate the software and answered any questions on how to use it.

**Table 1:** HappyNeuron® exercises used in Cog-Tab-Age study

**Memory (verbal and visual)**
Words, Where are you? Elephant Memory Shapes and Colors Heraldry Displaced Characters Displaced Images N-Back Around the World in 80 trips I Remember You! Restaurant An American in Paris Find Your Way! Chunking Objects, Where are You?
**Attention / Executive functions / Information processing speed**
Pay Attention! Private Eye! Sound Check! Ancient Writing Towers of Hanoi Basketball in New-York Hurry for Change! Two-Timing Under Pressure Gulf Stream Catch the Ladybug!
**Language**
Split words Embroidery Secret files Speak Your Mind! Decipher Writing in the Stars This Story is Full of Blanks! Which One is Alike?
**Logic**
The Right Count Countdown Ready, Steady, Count!
**Visuospatial abilities**
Sleight of Hands Entangled Figures Point of View Turn Around and Around

For the second and third session, the participants completed the exercises in autonomy (i.e. without the neuropsychologist). They had the option to perform sessions at home or at the cancer center. They were advised to use the software for 20 min and to choose the exercises themselves and the level of difficulty.

### Assessments

#### Baseline assessment

Demographic variables included age, family status, living status, help at home, level of education and previous experience with digital tools (assessed through the following question: “have you already used a smartphone or tablet?” with a multiple choices answer: never/sometimes/often). Clinical characteristics included cancer stage, previous cancer history, comorbidities, functional status with the Eastern Cooperative Oncology Group (ECOG) scale, prior and current anti-cancer therapies, and use of psychotropic medication.

Subjective cognitive complaints were assessed at baseline using the French version of the self-report Functional Assessment of Cancer Therapy-Cognitive Function (FACT-Cog) scale [30, 31], composed of four subscales: Perceived Cognitive Impairments (PCI), Impact on Quality of Life (QoL), Comments from Others (Oth), and Perceived Cognitive Abilities (PCA).

#### Assessments during intervention

Information collected in the second and third sessions performed in autonomy included the participants’ preference for onsite or at-home session, time spent navigating the interface (excluding time taken to complete exercises), number of exercises completed for each session, the cognitive domains most frequently trained, and interval between sessions.

#### Post-intervention assessment

Satisfaction with the software was assessed by a 9-item self-report questionnaire designed for this study. Satisfaction was rated on a 5-point Likert scale: “strongly disagree”, “disagree”, “neutral”, “agree”, and “strongly agree”. The questionnaire included questions concerning patients’ appreciation of the software: overall satisfaction, easiness and content of exercises, easiness of use of the software, need for more practice to feel comfortable with the software, interest to further use the software, and recommendations of the intervention to other people of similar age. The questionnaire also contained an open-ended question concerning their greatest challenges using the software.

### Study endpoints

#### Primary endpoint: usability

The 20 min spent using the tablet in the independent sessions were divided into “training time”, which was spent completing the exercises and was collected by the software, and “navigation time”, which was spent navigating the software and reading the exercise instructions, calculated by subtracting the training time from the 20-minute session. Based on our experience with the software, we hypothesized that an elderly patient would be able to spend half of the 20 min with the tablet, navigating the software, choosing the exercises etc. Thus, the usability was defined as completing at least three exercises during the training time (10 min) during one of the two independent training sessions.

If more than three exercises were completed, the software was considered to have a very high level of usability. Completion of three exercises corresponded to a high level of usability, two to a medium level, and one to a low level. Uncompleted exercises were considered as a very low level of usability of the software.

#### Secondary endpoints: eligibility, adherence and satisfaction

Eligibility to the digital cognitive intervention was defined as the proportion of patients meeting eligibility criteria from the total number of patients who were screened.

The level of patients’ adherence was determined by their completion of three sessions of the complete intervention (high adherence), two sessions out of three (medium adherence), or one session out of three (low adherence). Patients were considered as satisfied if they answered “agree” or “strongly agree” to questions in the satisfaction questionnaire.

### Statistical considerations

Assuming an acceptance rate of 70% with a 95% confidence interval of width (0.25), 49 assessable patients were required to meet the main criterion of evaluating the usability of the intervention. Anticipating a maximum of 10% of non-evaluable patients, we decided to enroll 55 patients.

Descriptive statistics were performed for the sociodemographic and clinical variables, mean and standard deviation for continuous variables and frequency with corresponding percentage for categorical variables. Reasons for non-eligibility and their corresponding percentage were reported, along with rate of eligibility, rate of acceptability and reasons for refusing to participate in the study. Frequency and percentage for each level of usability were reported. Comparisons were made with Fisher’s exact test for categorical variables and the Kruskal-Wallis rank sum test for qualitative variables.

If participants completed more than the two required sessions, only the required sessions were included in the analysis. For patients who had performed more than 20 min in one session, only the first 20 min were considered. Significant cognitive complaints were defined using the normative data of the PCI (Perceived Cognitive Impairment) subscale of the FACT-Cog self-report questionnaire (≤ 10th percentile of normative data depending on age [31]).

## Results

### Eligibility and acceptability of cognitive intervention

**Fig. 1:**
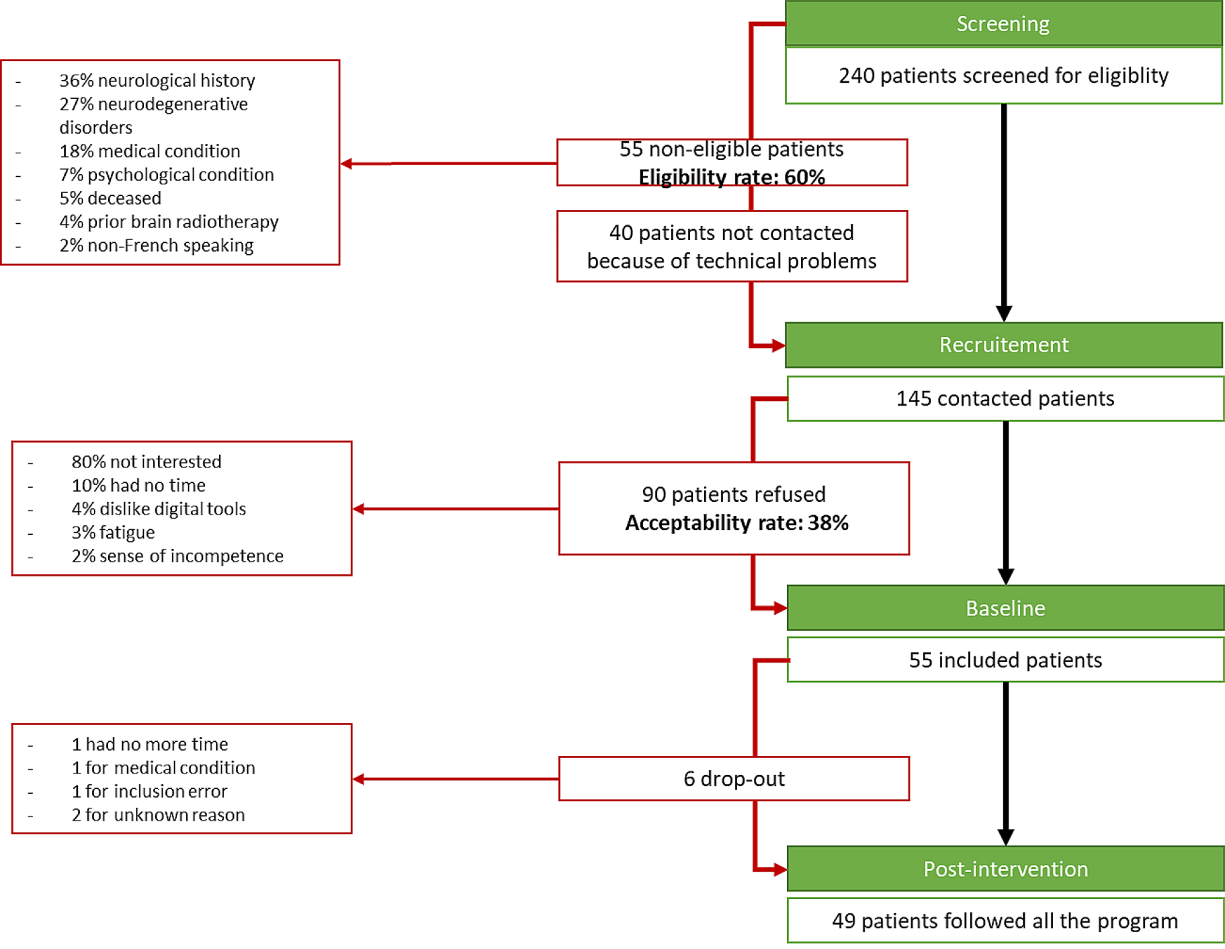
Flowchart of the study

Of the 240 patients with breast cancer and 70 years old or over, screened for eligibility between June 2020 and August 2022, 55 were ineligible (23%) (Fig. 1). The main reasons for ineligibility were neurological comorbidities (*n* = 20, 36%) and cognitive impairment based on MoCA score (*n* = 15, 27%). Forty patients (17%) could not be contacted because of organizational problems, such as changes in the treatment plan, problems in organizing their recruitment, and Covid-positivity. Overall, the eligibility rate was 60% of the screened patients. Of the 145 eligible patients who were contacted to participate, 90 refused (62% of eligible patients), mainly because they were not interested in participating in a study (80%). The acceptability rate was therefore 38% (Fig. 1), with 55 eligible patients agreeing to participate. Six participants dropped out, so the post-intervention assessment was performed in 49/55 patients (Fig. 1). Fifty participants completed at least one independent session second and/or third session, so the usability analysis was performed on this sample.

### Patient characteristics

Among the 55 patients included (mean age: 73 ± 3 years), most patients (96%) had an ECOG performance status ≤ 1 (Table 2). Current treatments were mainly radiotherapy (*n* = 35, 64%) chemotherapy (*n* = 26, 47%) and targeted therapy (*n* = 20, 36%) for stage I-II breast cancer (*n* = 43, 79%). 82% (*n* = 45) of patients had significant cognitive complaints (FACT-Cog, PCI) and the mean MoCA score was 27 (± 3). More than half of them (*n* = 30, 55%) had previous experience with digital tools.

**Table 2:** Participant characteristics (*N* = 55)

Characteristics	*N* = 55	%
Mean age± SD (range)		
	73 ± 3 (71–76)	
Family status		
Married/in couple	32	58
Widowed	14	26
Divorced	4	7
Single	5	9
Living status		
Alone	18	35
Not alone	34	65
Help at home		
Yes	26	55
Education		
Primary school	16	29
Middle school	19	35
High school	10	18
University	9	16
Unknown	1	2
Cancer stage		
I	2	4
II	41	75
III	9	16
Metastasis	6	11
Unknown	3	5
Comorbidity		
Yes	48	87
Functional status (ECOG)		
0	26	48
1	26	48
2	2	4
Prior anticancer therapies		
Surgery	52	95
Chemotherapy	15	27
Hormonal therapy	11	20
Radiotherapy	20	36
Anticancer therapy at baseline		
Targeted therapy	20	36
Chemotherapy	26	47
Radiotherapy	35	64
Hormonal therapy	9	16
Psychotropic medication		
Yes	12	22
Cognitive complaints (FACT-Cog - PCI)		
PCI score ≤ 10th percentile of normative data depending on age	45	82
Score MoCA (range)		
	27 (25–28)	
Previous experience with digital tools		
Often	22	40
Sometimes	8	15
Never	8	15
Unknown	17	0

### Usability of intervention and adherence

**Fig. 2:**
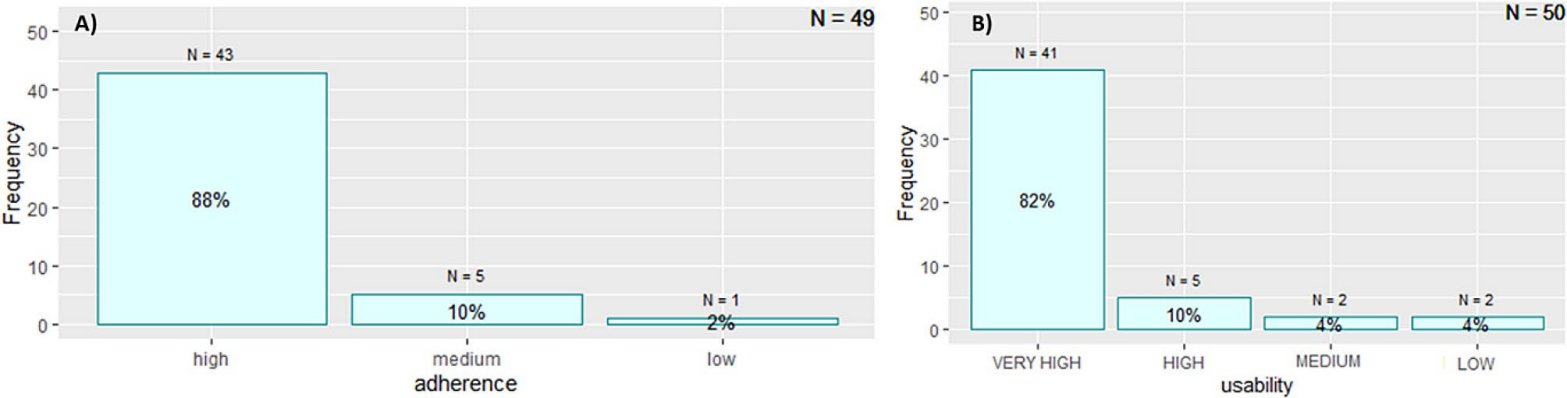
Feasibility of the intervention based on adherence and usability. (**A**): Proportion of participants who have completed 3 sessions (80%), two sessions (13%) and one session (7%). (**B**): Level of usability of the software during the two intdipendent sessions, calculated by the number of exercises completed in 10 min of using the software. 96% of participants completed more than 3 exercises in 10 min and 4% completed less than 1 exercises in 10 min

Ninety-two percent (*n* = 46/50) achieved the main objective of usability by completing at least three exercises. The level of usability was very high for 82% of participants (*n* = 41/50), with patients completing more than three exercises during the training time in one of the two independent training sessions, high for 10% of participants (*n* = 5/50) (three exercises completed), medium for 4% (*n* = 2/50) (two exercises completed) and low for 4% (*n* = 2/50) (only one exercise completed) (Fig. 2).

A high level of adherence was achieved by 88% (*n* = 43/49) of patients, who completed the three sessions of the cognitive intervention. 10% (*n* = 5/49) achieved a medium level of adherence and 2% (*n* = 1/49) achieved a low level of adherence (Fig. 2).

### Intervention characteristics

**Fig. 3:**
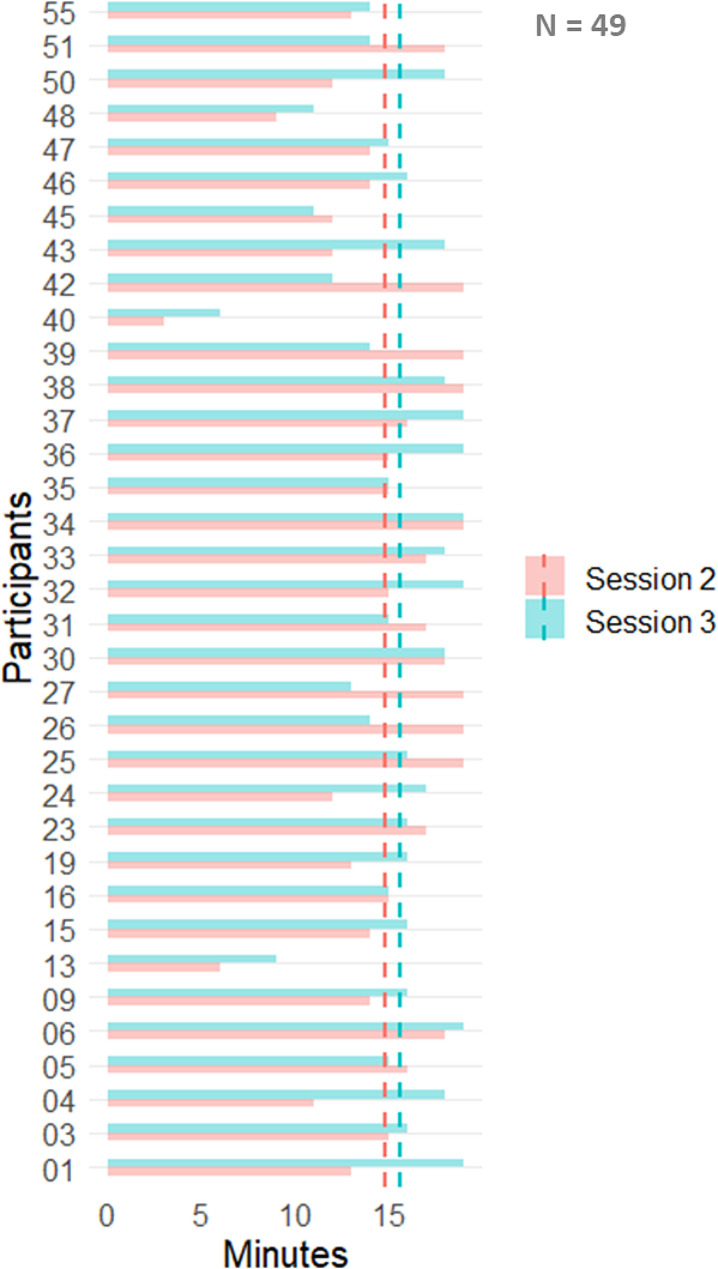
Participants’ navigation time during the second and third sessions. Pink and light-blue dot lines represent mean navigation time in session two and three, respectively

**Fig. 4:**
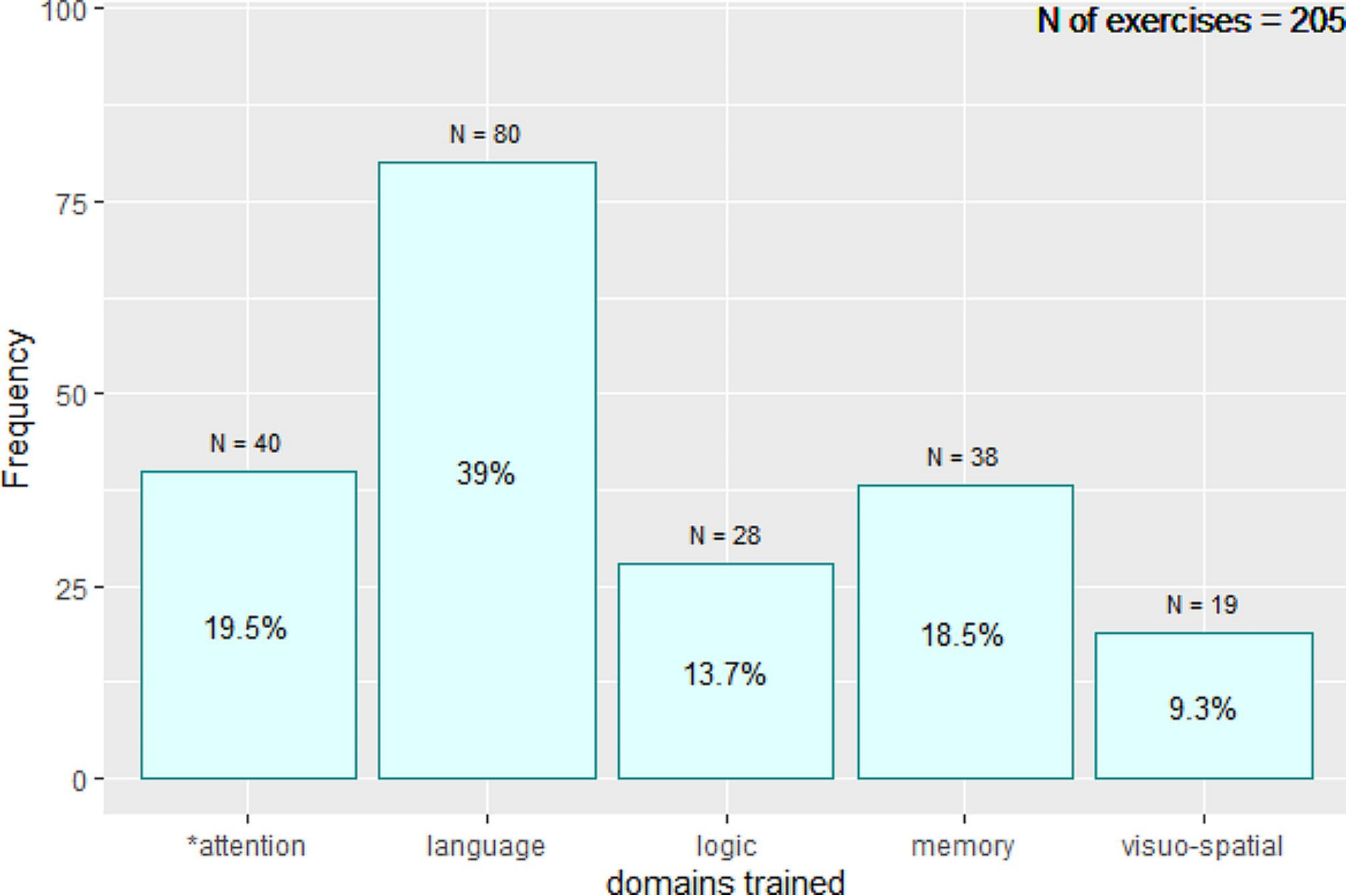
Cognitive domains trained during independent sessions Proportion of exercises completed by participants in session two and three divided by the domain trained *attention: attention / executive functions / information processing speed

Ninety percent of participants preferred the supervised on-site intervention to an unsupervised intervention at home. During the 20-minute session in autonomy, participants spent an average of 15 min during the second session and 16 min during the third session navigating the software interface and reading the exercise instructions (navigation time) (Fig. 3). Thus, the average time spent in training time was 5 min. On average, participants completed nine exercises in the second session and 10 in the third session. During the two sessions in autonomy, the cognitive domains most frequently trained were: language (39% of patients), attention/executive function/processing speed (20%), memory (19%), logic (14%) and visuo-spatial domain (9%) (Fig. 4). Overall, cognitive domains trained did not differ between the second and third session. The interval between the first and second session was on average 15 days, and between session two and three was 14 days. The maximum interval was 105 days.

### Associated factors

There was no significant influence of age, education, MoCA score, previous experience with digital tools, and ongoing treatments on usability, number of exercises completed and navigation time (data not shown).

### Participants’ satisfaction

**Fig. 5:**
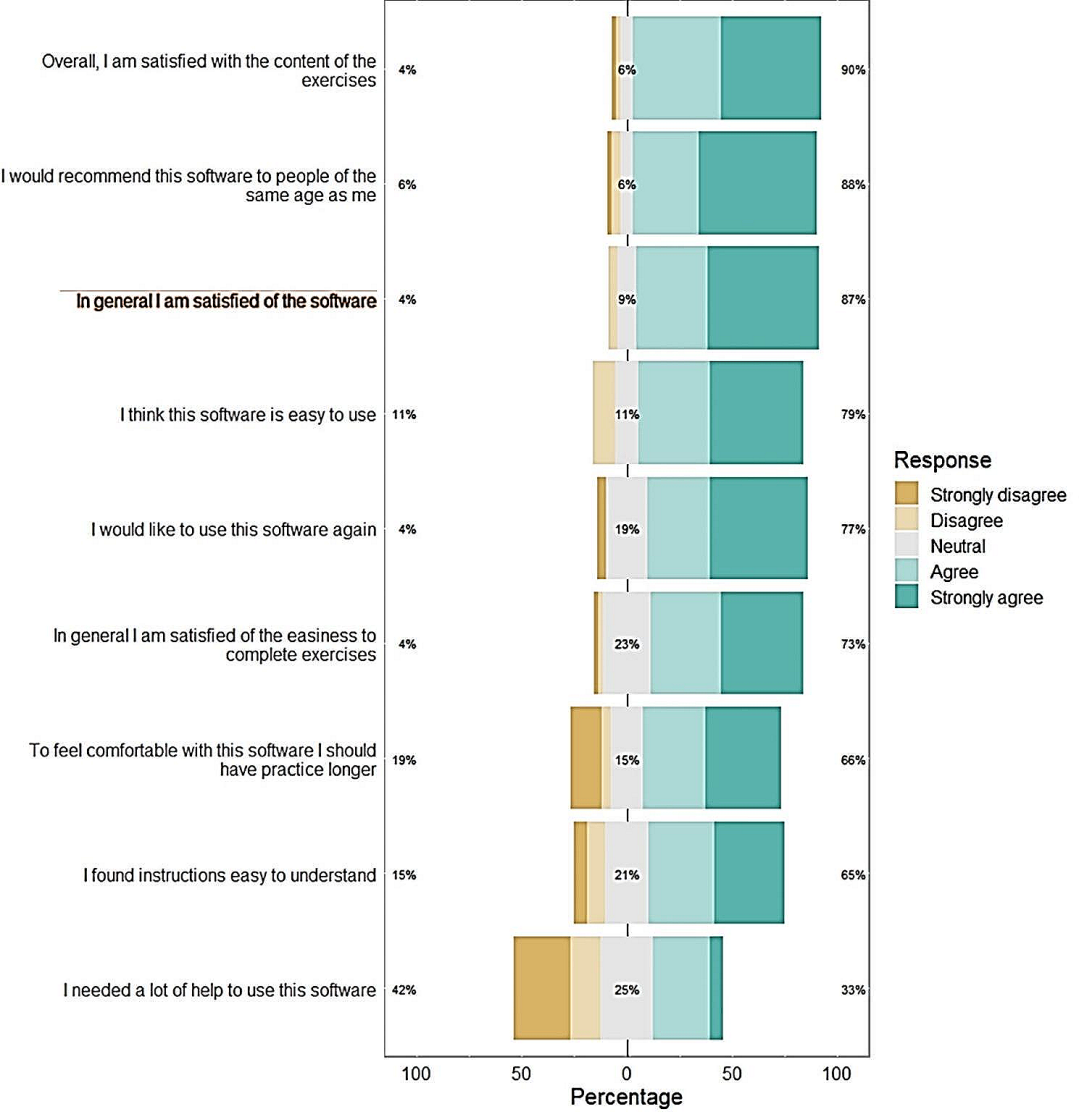
Satisfaction questionnaire results (5-points Likert scale)

**Fig. 6:**
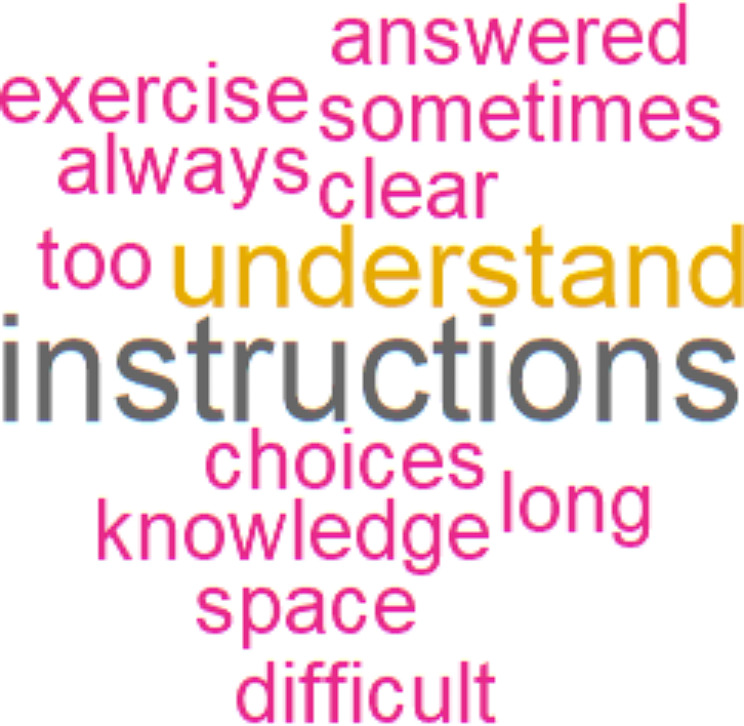
English translated world-cloud showing the most used words in the open-ended answers concerning difficulties encountered during the use of the cognitive stimulation software (question in the satisfaction questionnaire)

Eighty-seven percent of participants were satisfied overall by the software (*n* = 41/47). More than 70% were satisfied concerning: easiness of use, interest to use the software again, recommendation for people of the same age to use the software, easiness to perform the cognitive exercises, and the content of exercises (Fig. 5). Two thirds of participants felt the need to practice longer in order to feel comfortable with the software (*n* = 31/47, 66%). Most patients found the instructions easy to understand (*n* = 31/48, 65%), and one third felt that they needed a lot of help to use the software (*n* = 16/48, 33%). The word cloud text analysis performed on the open-ended answers concerning difficulties encountered during the use of the software in the satisfaction questionnaire showed the prevalence of the words: “instructions” and “understand” (Fig. 6).

## Discussion

This is the first study to show the feasibility of digital cognitive stimulation in elderly breast cancer patients older than 70 years. The participants were mostly capable of completing the cognitive exercises (92% managed to complete at least three exercises), and demonstrated a high level of adherence (88%) and satisfaction (in 87% of cases). The acceptability rate (38%) suggests that a more targeted approach is needed when selecting the population for this type of intervention. A possible solution could be to invite only patients with cognitive complaints to participate.

### Comparison to prior work

Our study is the first to investigate acceptability, usability, and adherence with regard to digital cognitive stimulation on elderly breast cancer patients older than 70 years. To our knowledge, only one previous study conducted by Wu et al. [21] explored similar parameters in 60 prostate cancer patients using the BrainHQ software. The rate of acceptability and adherence was 50% and 60%, respectively, with participants completing at least 10 h of training and experiencing a high level of satisfaction (mean score of 2.96 on a 3-point Likert scale). In our study, the acceptability rate was lower (38%), perhaps due to differences in the characteristics of the two populations. For example, the average age of our participants was 73 years, whereas average age was 66 years in the other study. Additionally, our participants were included regardless of their cognitive abilities, whereas Wu et al. included individuals with mild cognitive or neurobehavioral impairment. Focusing only on patients with cognitive impairment may have contributed to a slightly higher acceptance rate in their study. Accordingly, 82% of our participants reported significant cognitive complaints (score on the PCI subscale of the FACT-Cog ≤ the 10th percentile, according to age), suggesting that the intervention may be more acceptable to patients with cognitive complaints. This finding is consistent with the “Diffusion Theory” proposed by Atkin et al., which posits that the adoption of a technological innovation by elderly people is influenced by individual characteristics such as health, beliefs about the technology’s usability, and its perceived usefulness [32].

Our rate of adherence was 80%, whereas it was 60% in the Wu et al. study [21]. This difference might be due to the fact that they aimed to assess the feasibility of an eight-week intervention with at least 10 h of cognitive stimulation, while we evaluated patients’ ability to use digital cognitive stimulation software with only three sessions of 20 min, which is not typical of a cognitive stimulation program. Our aim was to assess the usability of a digital cognitive tool and not the program itself.

### Implementation of intervention: possible barriers and facilitators

We explored the factors that potentially help or hinder the implementation of interventions for cognitive rehabilitation in elderly breast cancer patients in a healthcare setting. Interestingly, the digital format of the intervention did not appear to be a major reason for declining participation, with only 4% of contacted eligible patients not accepting to participate for this reason. Instead, lack of interest in participating in a study was reported as the primary reason for declining participation (80%) resulting in a low participation rate (38%). We can hypothesize that among the patients whose participation was refused for this reason, there were patients with a low level of digital skills. Although it has been suggested that the elderly often avoid using digital tools owing to a lack of familiarity and access [33], having to use a digital tool did not deter most of our elderly patients, or was not explicitly mentioned, from participating in a cognitive stimulation program.

Regarding the preference for supervision or autonomy use, most patients (90%) preferred to complete the sessions at the cancer center under the supervision of the neuropsychologist. This finding suggests that despite being able to using digital tools (96% of participants based on the usability criteria) and completing cognitive training on a tablet, elderly individuals may not feel enough confident to do so without supervision, indicating a lack of familiarity with the technology. Supervision by a professional has already been identified as a facilitator for adherence and effectiveness of digital interventions in younger participants [23, 24]. Furthermore, elderly participants are known to be diffident about the lack of human contact in remote digital interventions [34]. In addition, being supervised could reduce the level of anxiety that often experienced by elderly participants when using unfamiliar technology [35].

Supervision by a professional and preparing the session (could also maximize the usefulness of the intervention and allow patients to use the given time for training only). When our patients used the software alone, they spent an average of 15–16 out of 20 min navigating the software and reading the instructions. This indicates that only a few minutes were dedicated to cognitive training, so it is possible that they experienced some difficulties navigating the software. Despite the excessive time spent navigating the software, participants felt that the program was easy to use and suitable for individuals of their age (88%).

Among the main trained cognitive domains, participants exhibited a preference for exercises that targeted language (39%), attention/executive functions/information processing speed (20%), and memory (19%). This is likely because these domains are among the most affected in patients with CRCI, and participants probably chose exercises that focus on these areas. Moreover, these exercises had a more amusing content, which may have attracted more attention from the participants.

### Limitations

This study has some limitations. First, information on patients’ preferences for digital or non-digital interventions was not collected. Second, the study was proposed to all patients without eligibility criteria for cognitive complaints, resulting in a low acceptance rate that may not reflect the population of interest, i.e. elderly breast cancer patients with CRCI. Third, the study was conducted during the COVID-19 pandemic, which posed additional challenges for patient recruitment. Lastly, the results do not represent a standardized intervention and on satisfaction and usability may be specific to the software used.

### Future directions

This study represents an initial exploration of elderly patients’ preferences and needs for interventions for CRCI. As a preliminary investigation, this feasibility study does not represent a model of a potential intervention to be implemented in clinical practice. Further studies should deepen the understanding of preferences for intervention modalities, exploring the feasibility of each modality for elderly patients. Specifically, further studies should focus on determining the optimal frequency and duration of sessions, the total duration of the intervention and the most appropriate timing for intervention proposals.

Our results indicate a clear preference of elderly patients for supervised cognitive interventions. Nevertheless, in clinical practice, most clinics do not have a neuropsychologist to supervise the intervention [35]. Other trained staff could be involved but they require dedicated space and time. An alternative might be to propose longer training period for elderly patients or remote supervision, by phone or videoconference, but its acceptability and feasibility among elderly patients needs to be investigated.

In this study, we also noted that patients showed a preference for specific exercises. For a deeper understanding, future research should assess the reasons behind participants’ choices and preferences. This exploration may also highlight potential barriers or facilitators to patient motivation, providing valuable information for future research.

Finally, future studies should aim to compare various interventions (i.e. physical activity and cognitive stimulation) in terms of their effectiveness and feasibility.

## Conclusion

The high level of usability, adherence, and satisfaction in this study demonstrates for the first time the feasibility of digital cognitive stimulation in patients with cancer who are older than 70 years. However, the intervention should be proposed only to patients reporting cognitive complaints and should be structured and supervised to result in better acceptability and adherence. Further research is needed to investigate the feasibility and efficacy of digital interventions in addressing CRCI in the elderly.

## Data Availability

The datasets used during this study are available from the corresponding author upon reasonable request.
